# The effect of platelet-rich plasma on osseous healing in dogs undergoing high tibial osteotomy

**DOI:** 10.1371/journal.pone.0177597

**Published:** 2017-05-16

**Authors:** Samuel P. Franklin, Emily E. Burke, Shannon P. Holmes

**Affiliations:** 1 Department of Small Animal Medicine and Surgery, University of Georgia, Athens, Georgia, United States of America; 2 Regenerative Bioscience Center, University of Georgia, Athens, Georgia, United States of America; 3 Veterinary Biosciences and Diagnostic Imaging, University of Georgia, Athens, Georgia, United States of America; University of Pisa, ITALY

## Abstract

**Objectives:**

The purpose of this study was to investigate whether platelet-rich plasma (PRP) enhances osseous healing in conjunction with a high tibial osteotomy in dogs.

**Study design:**

Randomized controlled trial.

**Methods:**

Sixty-four client-owned pet dogs with naturally occurring rupture of the anterior cruciate ligament and that were to be treated with a high tibial osteotomy (tibial plateau leveling osteotomy) were randomized into the treatment or control group. Dogs in the treatment group received autologous platelet-rich plasma activated with calcium chloride and bovine thrombin to produce a well-formed PRP gel that was placed into the osteotomy at the time of surgery. Dogs in the control group received saline lavage of the osteotomy. All dogs had the osteotomy stabilized with identical titanium alloy implants and all aspects of the surgical procedure and post-operative care were identical among dogs of the two groups. Bone healing was assessed at exactly 28, 49, and 70 days after surgery with radiography and ultrasonography and with MRI at day 28. The effect of PRP on bone healing was assessed using a repeated measures analysis of covariance with radiographic and ultrasonographic data and using a t-test with the MRI data.

**Results:**

Sixty dogs completed the study. There were no significant differences in age, weight, or gender distribution between the treatment and control groups. Twenty-seven dogs were treated with PRP and 33 were in the control group. The average platelet concentration of the PRP was 1.37x10^6^ platelets/μL (±489x10^3^) with a leukocyte concentration of 5.45x10^3^/μL (±3.5x10^3^). All dogs demonstrated progressive healing over time and achieved clinically successful outcomes. Time since surgery and patient age were significant predictors of radiographic healing and time since surgery was a significant predictor of ultrasonographic assessment of healing. There was no significant effect of PRP treatment as assessed radiographically, ultrasonographically, or with MRI.

**Conclusion:**

The PRP used in this study did not hasten osseous union in dogs treated with a high tibial osteotomy.

## Introduction

Improving the rate and effectiveness of osseous healing after fracture, bone loss, or osteotomy remains a clinically relevant goal in orthopedics.[1, 2] Anabolic growth factors such as transforming growth factor β-1, vascular endothelial growth factor, and platelet-derived growth factor are contained in abundance within platelet alpha granules and their delivery to fracture or osteotomy sites could theoretically stimulate osseous regeneration.[3, 4] As a result, platelet-rich plasma (PRP) has commonly been investigated for its role in augmenting bone regeneration in both preclinical models and clinical studies in people. Multiple reviews have been performed of the available data.[5–8] Many data from pre-clinical models provide support for the benefits of PRP in augmenting osseous union and some authors conclude there is sufficient evidence to demonstrate proof of principle.[8] However, other authors conclude that the evidence remains ambiguous as numerous studies, particularly when considering clinical trials in people, fail to provide consistent or convincing evidence of benefit.[7] Contradictory results from different studies and contradictory conclusions from different reviews preclude making a general conclusion as to whether PRP is likely to augment bone healing.

Some of the possible reasons for discrepant results include investigation in different species, use of PRP with different bone defect or fracture models, use of different PRP products, and use of different outcome measures to assess efficacy. All of these variable have likely contributed to the extreme heterogeneity in the data available.[7] As a result, further research is needed to determine which specific types of PRP may be beneficial in improving bone healing and in which specific medical conditions. In order to address this issue there is a need for well-controlled experiments using thoroughly characterized PRP products, including in preclinical animal models.

**Fig 1:**
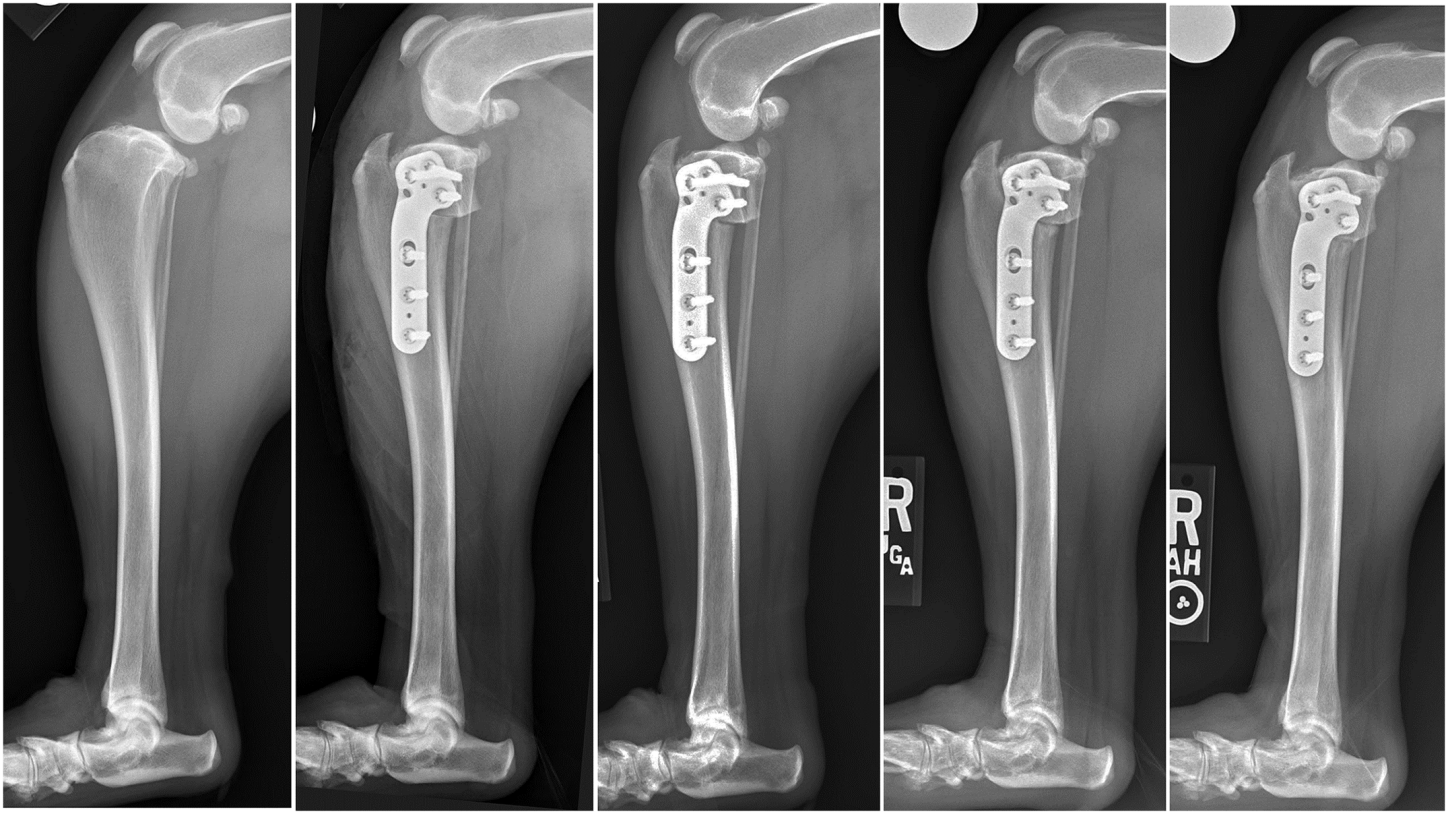
Representative medial-lateral radiographs for one dog pre-operatively, immediate post operatively, and 4, 7, and 10 weeks post operatively. Caudal femoral subluxation is apparent on the pre-operative medial-lateral view and is resolved on all subsequent medial-lateral views. The bone plate obscures approximately 50% of the osteotomy on the medial-lateral radiographic view. However, progressive radiographic healing can be seen anterior and posterior to the bone plate.

**Fig 2:**
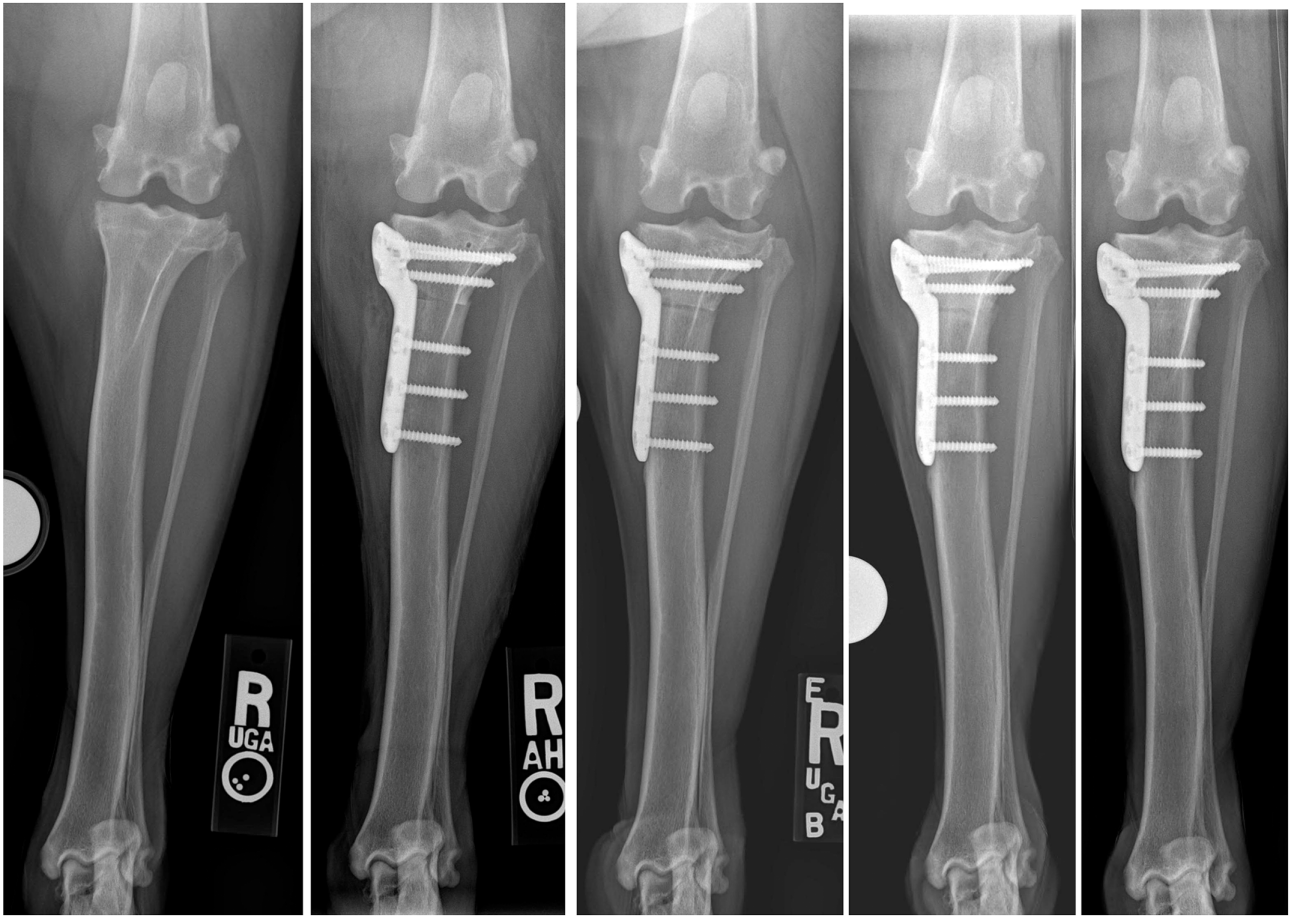
Representative posterior-anterior radiographs for the same dog as in Fig 2 pre-operatively, immediate post operatively, and 4, 7, and 10 weeks post operatively. Progressive radiographic healing of the osteotomy can be seen.

Treatment of anterior cruciate ligament (ACL) rupture with osteotomy in client-owned pet dogs provides a potentially useful and unique opportunity for assessing the effects of PRP on bone healing. Naturally-occurring rupture of the (ACL) is extremely common with one report estimating that 1.2 million surgeries are performed each year in the USA to treat ACL rupture in dogs.[9] High tibial osteotomy, and more specifically tibial plateau leveling osteotomy (TPLO), is one of the most commonly performed surgeries to treat dogs with ACL rupture.[10] This surgery involves performing a metaphyseal rotational osteotomy to decrease the tibial plateau angle (posterior tibial slope), thus minimizing femorotibial subluxation and helping dogs cope with being ACL-deficient (Figs 1 and 2).[11] Although results with this procedure are usually good, complications can include delayed osseous union, nonunion, and mechanical failure with loss of reduction prior to attainment of osseous union, similar to HTO in human patients.[12–14] Accordingly, if PRP were effective in enhancing the rate of osseous union it would have clinical utility for dogs treated with TPLO. In addition, should PRP augment bone healing in dogs treated with TPLO, a human PRP with similar characteristics would be worthy of investigation for its potential application in people.

The purpose of this study was to investigate the utility of a well-characterized PRP for enhancing osseous union in dogs treated with TPLO using multiple clinically-applicable outcome measures including radiography, ultrasonography, and magnetic resonance imaging (MRI). We hypothesized that dogs treated with PRP would have more rapid healing of their osteotomy as assessed with radiography and MRI than dogs not treated with PRP.

## Methods

### Study subjects

Sixty-four client-owned pet dogs with naturally-occurring rupture of the ACL were enrolled in this prospective, randomized, blinded clinical trial. This study was approved by the Clinical Research Committee at the University of Georgia and all owners provided written consent for enrollment of their dogs. Enrollment criteria included that the dog needed to be diagnosed as having ACL rupture based upon orthopedic examination and pre-operative radiography as is routinely performed for dogs with ACL rupture. Further, all dogs were between 2 and 10 years of age and weighed at least 25 kilograms. All dogs needed to have a tibia that was of appropriate size to be treated with a 21 mm radius TPLO saw blade and stabilized with a 6-hole custom 3.5 TPLO plate. All dogs were free of any other medical problems and were not receiving any medications at the time of TPLO.

Enrolled dogs were randomly assigned to either the treatment (PRP) or control (saline) group using a random number generator. All dogs were anesthetized using the same anesthetic medication protocol including pre-medication with 0.005 mg/kg body weight of dexmedetomidine (Zoetis, Florham Park, NJ) and 0.1 mg/kg of hydromorphone (Hospira Inc, Lake Forest, IL). Anesthesia was induced with 5 mg/kg of Ketamine (Akorn, Lake Forest, IL) and 0.25 mg/kg of diazepam (Hospira, Lake Forest, IL) and maintained with Isoflurane (Piramal, Bethlehem, PA).

Once anesthetized, and immediately preceding surgery, an 18-gauge 2-inch intravenous catheter was aseptically placed in a jugular vein for those dogs included in the PRP group. Fifty-two mls of blood was collected into two 60-ml syringes preloaded with 8mls of ACD-A to provide a total volume of 120 ml of anticoagulated blood. A 0.5 ml aliquot of this blood was mixed with ethylenediaminetetraacetic acid (EDTA) and then assessed to characterize the cellular concentrations in the whole blood using an automated hemacytometer (Element HT5, Heska Corporation, Loveland, CO). The sample was mixed with EDTA prior to assessment of the cellular composition as some evidence suggests that use of EDTA prior to analysis optimizes platelet enumeration.[15] The remaining anticoagulated blood was used to prepare PRP using a commercially available system (Angel System, Arthrex Vet Systems; Naples, FL) using the manual function and visually distributing the red blood cell, platelet-poor plasma, and PRP fractions. After preparation, an aliquot of the PRP was similarly mixed with EDTA and assessed using the same hemacytometer similar to previous work.[15]

After blood acquisition and while the PRP was being prepared, surgery was commenced including arthroscopic evaluation of the affected stifle and intra-articular structures including the ACL and menisci. Ruptured ACLs were debrided and torn menisci were treated with arthroscopic partial meniscectomy. Immediately following joint inspection the TPLO was performed. All surgeries were performed by the principal investigator and specific attributes of the surgical procedure were standardized for all dogs. These attributes include that no elevation of the soft tissue envelope from the posterior or lateral aspects of the tibia with packing of gauze was performed. No dogs had simultaneous correction of either torsional or varus/valgus malalignment. The same TPLO saw blade was used in all dogs and saline irrigation during osteotomy performance was consistent for all dogs. Standardized locking titanium bone plates and screws were used in all dogs.

**Fig 3:**
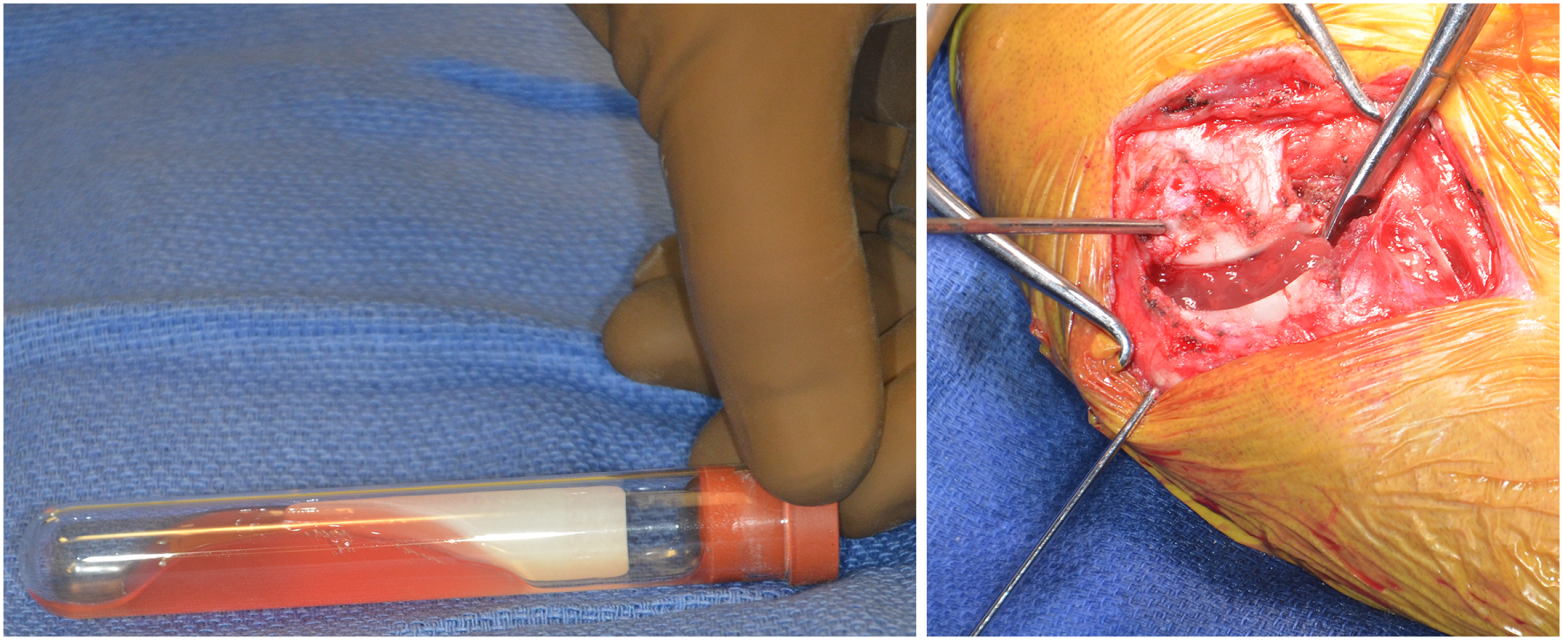
The activated PRP immediately after it has formed a malleable gel and with placement into the distracted osteotomy. Note that the osteotomy would be reduced and stabilized following distraction and PRP placement.

After the osteotomy was made it was distracted to enable placement of the activated PRP gel or lavage with saline (Fig 3). In order to create the PRP gel 5,000 IU of bovine thrombin (Thrombin-JMI^®^, Pfizer, New York) was re-constituted with 5 ml of 10% calcium chloride solution (Ansyr^™^, Hospira/Pfizer, New York) to create an activation solution. The PRP was then mixed with this solution in a 10:1 ratio based upon PRP volume (PRP:activation solution) to form a malleable PRP gel, also referred to as a platelet-rich fibrin matrix (Fig 3).[16] The PRP and activator were manually mixed in a sterile glass blood tube for approximately 10–30 seconds as gel formation proceeded. Approximately 90% or more of the PRP was subjectively incorporated within the fibrin gel with relatively little (less than 10%) liquid PRP releasate. The PRP gel was manually introduced into the osteotomy site at the time of surgery in all cases treated with PRP (Fig 3). The osteotomy site was distracted and irrigated with saline in control patients. The osteotomy was then reduced and stabilized using a commercially un-available titanium 6-hole locking TPLO plate (Arthrex Vet Systems, Naples, FL) and 1 cortical and 5 locking titanium screws (Figs 1 and 2). Compression of the osteotomy was performed using the single hole in the bone plate distal to the osteotomy that allows for placement of a cortical bone screw in the load position with 5 locking screws placed in the other holes; all screws were bicortical.

Following surgery, post-operative orthogonal posterior-anterior and medial-lateral tibial radiographs were made to confirm appropriate surgical performance. Radiographic positioning was standardized such that the patella was centered over the distal femur and the fabella were bisected by the lateral cortices of the femur for the posterior-anterior images. For the medial-lateral images the knee and tarsal joints were flexed to 90°, the femoral condyles and fabella were superimposed, and the intercondylar eminences of the proximal tibia were visible. Post-operative instructions and analgesic regime were strictly uniform for all patients with regard to several variables such as post-operative medications including that non-steroidal anti-inflammatory medications (Carprofen 4.4 mg/kg per day, Zoetis, Florham Park, NJ) were prescribed for exactly 7 days following surgery given that non-steroidal anti-inflammatory medications have been shown to influence bone healing.[17] All owners were provided with the same activity modification instructions following surgery.

### Outcome measures

Dogs were re-examined, anesthetized, and radiographic, ultrasonographic, and MRI evaluations were performed at exactly 28 days post surgery. The anesthetic protocol included pre-medication with 0.005 mg/kg body weight of dexmedetomidine and 0.1 mg/kg of hydromorphone and induction with 4 mg/kg of Propofol (Zoetis, Florham Park, NJ). Anesthesia was maintained with isoflurane. For radiographic examinations the limbs were positioned as mentioned above for the immediate post-operative radiographic examination. In addition, particular attention was given to the orientation of the implants (plates and screws) such that the orientation of the implants were similar at all radiographic examinations, thus ensuring that limb positioning was consistent over time. Dogs were subsequently re-examined, sedated, and radiographic and ultrasonographic evaluations were performed at exactly 49 and 70 days following surgery. Sedation was obtained using 0.005 mg/kg of dexmedetomidine and 0.5 mg/kg of nalbuphine (Hospira, Lake Forest, IL).

### Healing assessments

#### Radiography

Radiographic assessment of osseous healing was performed by one investigator (SH) who was blinded to group assignment. Radiographic scoring was based upon modification of two scoring systems previously used to assess TPLO healing.[18, 19] In one of those previous studies a 10-point scale was used including assessing cortical healing on a 0–2 subscale.[18] We modified this cortical healing subscale to 0–4 as we scored cortical healing of two cortices on each of the medial-lateral and posterior-anterior radiographic views. With this modification the maximum cortical bone healing subscale was 4 and the total maximum healing score could be 12. Another study that evaluated bone healing following TPLO graded healing from 0–4 (0% healed, 1–25% healed, 26–50% healed, 51–75% healed, and 76–100% healed) based upon a medial-lateral radiographic projection alone.[19] We used the same scoring system but graded both orthogonal radiographic views and calculated the mean of these 2 scores.

#### Ultrasonography

**Fig 4:**
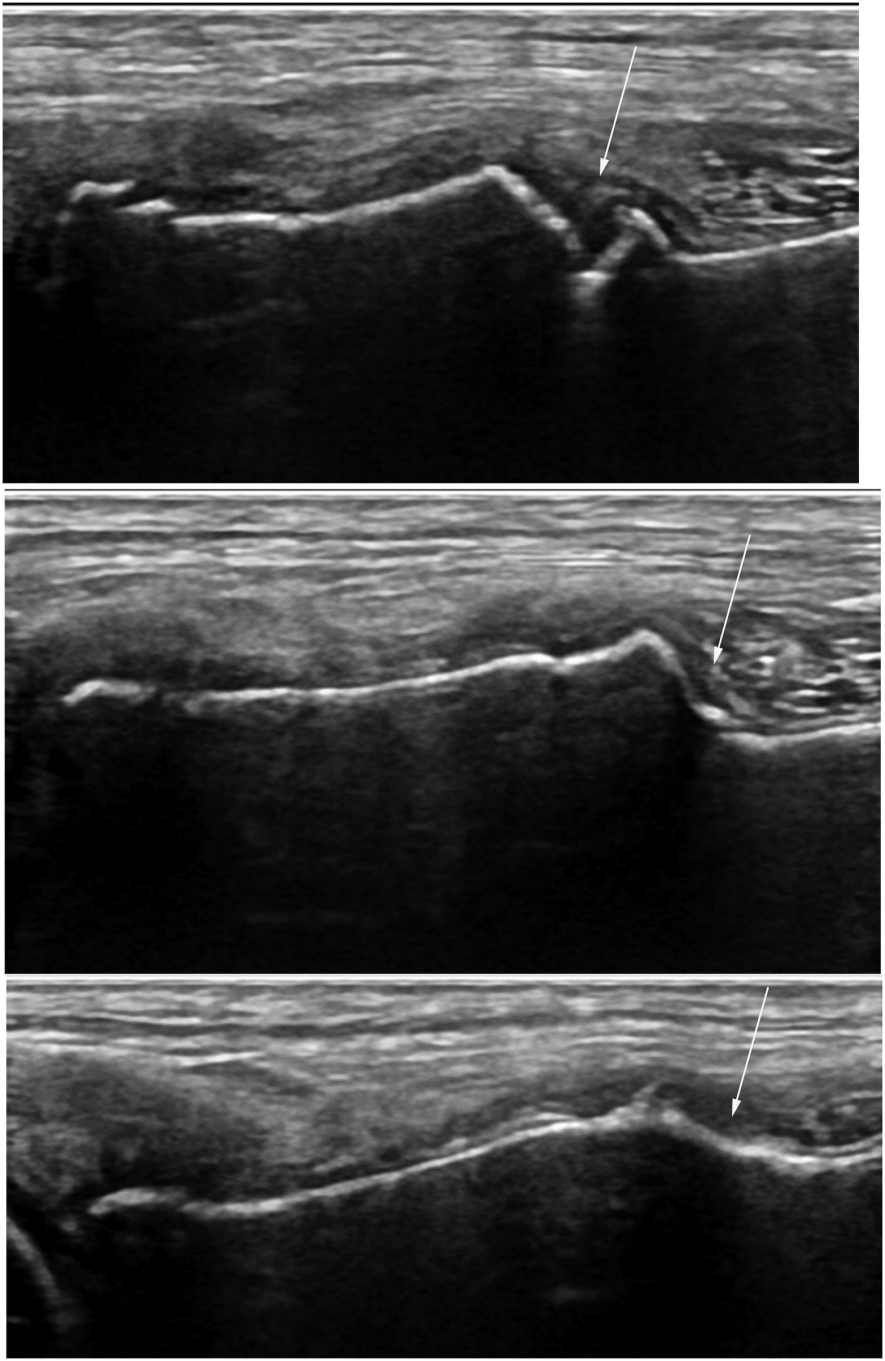
Brightness mode ultrasonographic images for one dog at 4, 7, and 10 weeks following surgery. Note the progressive healing of the osteotomy (arrows) over time.

**Fig 5:**
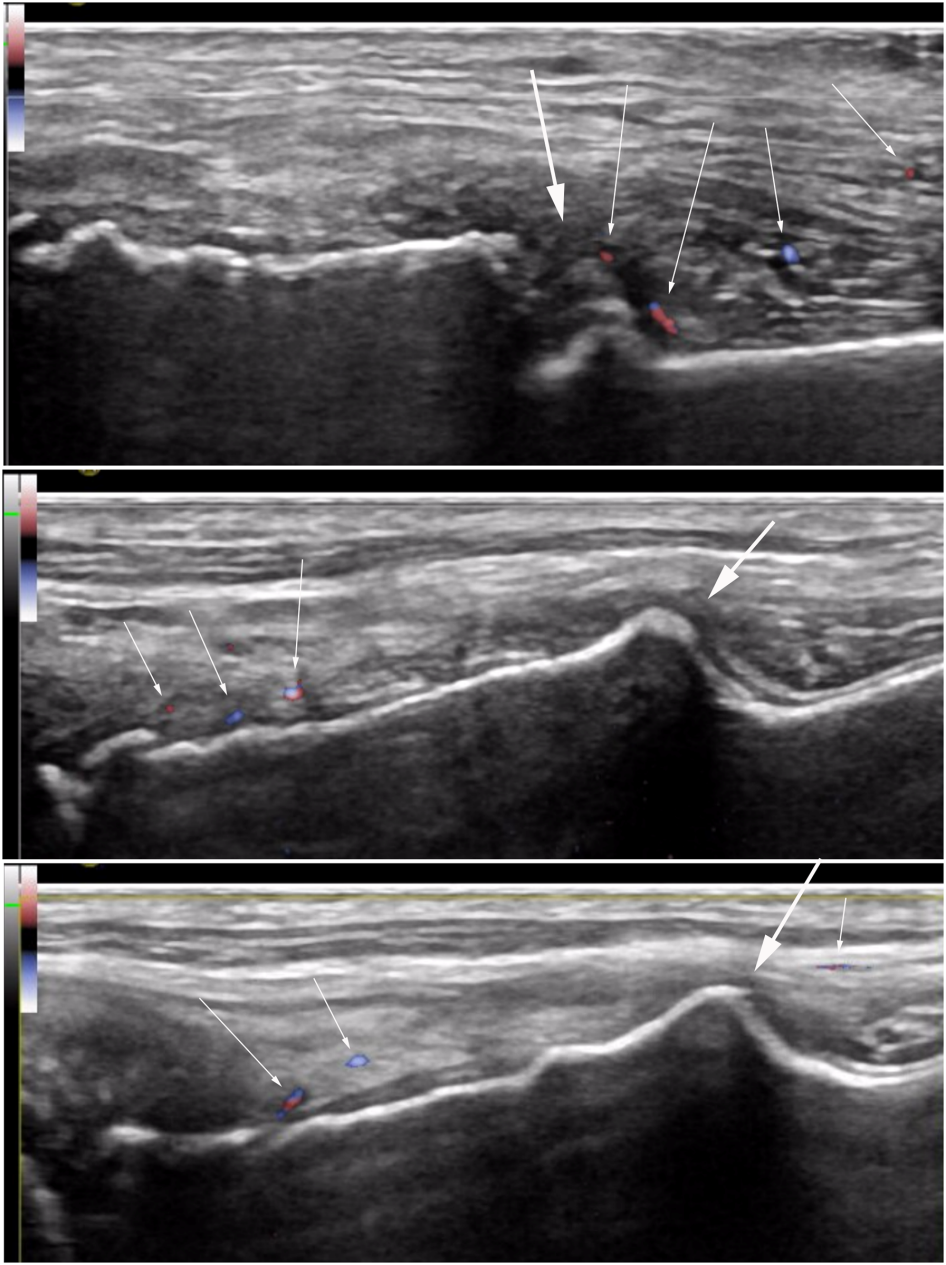
Power doppler ultrasonographic images for one dog at 4, 7, and 10 weeks following surgery. Note the decreasing vascular density over time (small arrows) corresponding to progressive healing of the osteotomy (larger arrows).

Ultrasonographic assessments of osseous healing were performed by one investigator (EB) using a portable ultrasonography unit (Noblus, Hitachi Aloka Medical, Ltd.) and a linear array transducer (L64, 18–5 MHz; Fig 4). Evaluation locations included the craniomedial, caudomedial, craniolateral, and caudolateral aspects of the proximal tibia and osteotomy. Still images and cine clips of the ultrasound exam were saved. At the conclusion of the study, all patient identification information was removed from the images and cine clips to mask group assignment to the reviewer (EB). Subsequently, the same ultrasonographer evaluated cortical continuity using B-mode ultrasonography and it was scored similarly to radiography, using a 0–4 scale with a score of four representing continuous cortices at all four of the aforementioned anatomic locations. Callus formation was also assessed using B-mode ultrasonography and graded on a 0–4 scale (0 = absent, 1 = minimal, 2 = moderate, 3 = remodeled, 4 = healed) using methodology that has previously been validated for use in dogs when assessing fracture healing.[20–22] Power Doppler ultrasonography was used to evaluate healing using a 0–3 scale, based upon the color and vascular density (area in mm^2^) as has been previously described for assessing canine fracture healing (Fig 5; Table 1).[23]

**Table 1:** Power Doppler ultrasonographic scoring scheme.

Category/score	0	1	2	3
**Color**	none	blue	red/purple	white
**Vessel area (mm**^**2**^**)**	none	<5mm2	5-10mm2	>10mm2
**Number**	none	<5	5 to 10	>10

#### MRI

**Fig 6:**
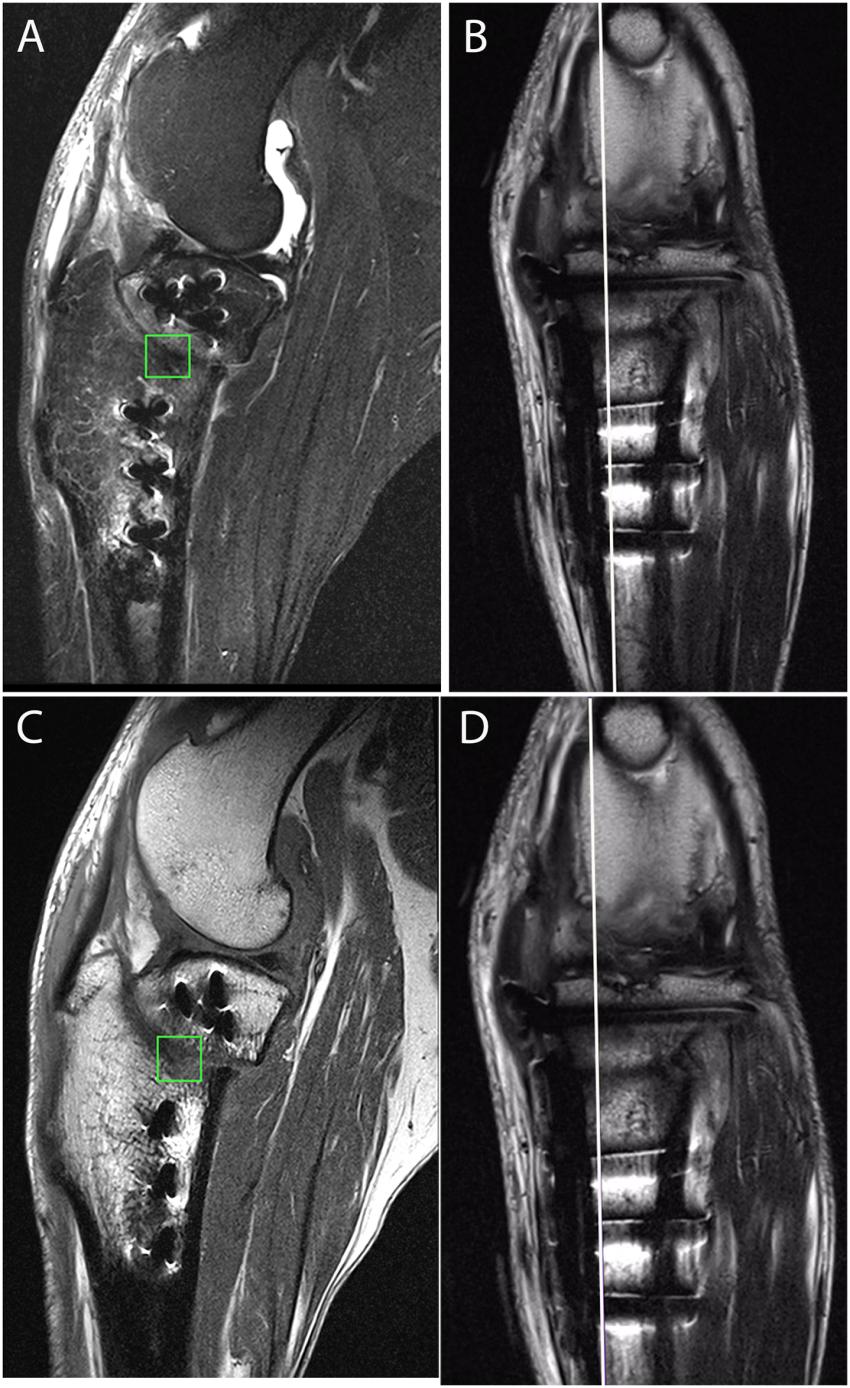
Representative MRI images used for quantitative assessment of osseous healing and showing placement of the square regions of interest on the sagittal slices and in which the intensity was measured. The corresponding white lines on the dorsal plane images demonstrate the location of the sagittal slice in which the ROI was drawn with regard to medial-lateral location. A) T2 Dixon fat suppressed sagittal plan sequence; B) Proton density weighted frontal plane sequence; C) T1 turbo spin echo sagittal plane sequence; D) Proton density weighted.

Dogs were placed in dorsal recumbency, with the affected leg placed in a knee coil (Siemens Tx/Rx 15-Channel Knee Coil) with the stifle flexed to about 120°. Images were obtained using a 3T MRI unit (Siemens Magnetom Skyra 3T, Erlangen, Germany) and using the parameters specified in Table 2 (Fig 6). Images were subsequently evaluated using Osirix version 6.5.2 (Bernex, Switzerland). The sagittal plane intermediate-weighted (IW) Dixon FS and dorsal plane T1-weighted (T1w) sequences were used simultaneously for region of interest (ROI) placement and to ensure no cortical or soft tissue partial volume averaging occurred within the ROI. Square ROIs (75mm^2^ area) were drawn on the sagittal plane images spanning the osteotomy (Fig 6). Values for the mean pixel intensity within the ROI were recorded. This process was repeated on 3 sagittal plane images that corresponded to the most medial, central, and most lateral portions of the proximal tibial medullary bone. Care was taken to ensure the most medial slice was not affected by perceptible metal artifact. The ROI evaluation of the T1w sagittal plane images was performed after the IW images using the same methods.

**Table 2:** MRI sequence parameters.

Parameters	T1 Turbo Spin Echo (TSE)	IW DIXON
**Image weighting**	T1	Intermediate
**Plane of acquisition**	Sagittal	Sagittal
**2D vs 3D**	2D	2D
**Echo time (TE)**	9.4	79
**Repetition time (TR)**	591	4530
**Flip angle**	180	120
**Number of acquisitions**	2	1
**Slide thickness**	1.6mm	2mm
**FOV and matrix dimensions**	100*140 FOV, 644*896 matrix	140*140 FOV, 768*768 matrix
**Imaging options**	WARP on	Fat-saturated
**Bandwidth**	445	310
**Sequence length**	3:09	9:49

### Statistical analysis

Age and body weight of dogs in the two groups were compared with unpaired t-tests. Gender distribution between the two groups was compared using a Fisher’s exact test. The effect of treatment on radiographic and ultrasonographic assessment of bone healing were evaluated in two ways. First, for each of the radiographic and ultrasonographic scoring systems, differences between the two treatment groups were compared at each time point using a one-tailed t-test and a p-value set at 0.05 *a priori*. These analyses were done with the radiographic 5-point and 12-point scales and the ultrasonographic assessments of cortical bone healing, callus formation, and power Doppler analysis. Similarly, evaluation of the MRI-assessed outcomes (IW Dixon and T1w TSE images) were assessed using one-tailed t-tests with a p-value set at 0.05.

Evaluation of the radiographic and ultrasonographic data were also performed using a repeated measures analyses of covariance. The initial models included the explanatory variables dog age, body weight, gender (all dogs were neutered except for one intact female dog and so all dogs were classified as just male or female), time (4, 7, 10 weeks), treatment (PRP or control), and the interaction terms treatment-by-time, weight-by-time, and age-by-time. Non-significant interaction terms were eliminated in a backward fashion if the p-value exceeded 0.75 until a final model was obtained.

## Results

Data were obtained and included in analyses for 60 dogs. All data from 4 dogs were excluded from statistical analyses because they did not return for follow-up (n = 1), had PRP that did not form a gel because it was activated with calcium chloride only and no thrombin was used in the activation (n = 1), had a fibular fracture recognized at the 4 week recheck (n = 1), or had an increase in tibial plateau angle (posterior tibial slope) from 5° to 13° at the 4 week re-evaluation consistent with mechanical instability (n = 1). Three of these dogs had PRP placed at the time of surgery and 1 of these dogs was in the control group, the dog that suffered fibular fracture. One dog had MRI data excluded from analysis, but radiographic and ultrasonographic data were included, because the MRI unit was dysfunctioning on day 28 post surgery and the values from the MRI analysis were dramatically different than values from all other 59 dogs.

There were no statistically significant differences in age (p = 0.15), body weight (p = 0.83), or gender distribution (p = 0.99) between dogs in the PRP and control groups (Table 3). Twenty-seven dogs were treated with PRP and 33 were included in the control group. Dog breeds represented were mixed breed (n = 31), Labrador retriever (n = 14), Golden retriever (n = 4), Pit Bull terrier (n = 3), Boxer (n = 3), and one each of American bulldog, goldendoodle, Husky, Rottweiler, and Springer Spaniel.

**Table 3:** Demographic data on dogs included in the study. **Measures of precision include the standard deviation followed by the range**.

	PRP group (n = 27)	Control group (n = 33)	p-value
**Age**	4.9 years (±1.7; 2.2–8.1)	5.7 years (±2.4; 2.0–10.2)	0.15
**Bodyweight**	32.2 kg (±5.4; 20.4–42.8)	31.9 kg (±4.7; 22.8–40.9)	0.83
**Male**	0	0	0.99*
**Intact Female**	0	1	
**Castrated Male**	12	15	
**Spayed Female**	15	17	

* The p-value corresponds to the comparison of gender distribution between the treatment and control group.

The prepared PRP had an average platelet concentration of 1.37x10^6^ platelets/μL (±489x10^3^), a leukocyte concentration of 5.45x10^3^/μL (±3.5x10^3^), and a negligible red blood cell concentration. This represented a mean 7.4X increase in platelet concentration above the baseline whole blood from which the PRP was prepared and a mean 1.1X increase in leukocyte concentration above the baseline whole blood sample. The average volume of PRP prepared and placed in the osteotomy was 4.9 mls. The mean ratio of platelet concentration to leukocyte concentration was 378 (±323).

All 60 dogs had successful outcomes based upon subjective assessment criteria previously established for veterinary patients.[24]. Successful outcomes were also obtained based upon in-house re-evaluation for the 3 dogs returned to our hospital for repeat examination and based upon phone interview for the one dog that was not returned for re-evaluation. All dogs had progressive healing over time based upon radiography (Tables 4 and 5) and ultrasonography (Tables 6–8). Radiographic scores for healing were numerically greater at all time points (4, 7, 10 weeks) for the PRP treatment group using the 5-point (Table 4) or 12-point scales (Table 5). Using the one-way t-test, there was significantly greater healing for the PRP treated group at 7 weeks (1.01 ± 0.6, p = 0.04) and at 10 weeks (0.94 ± 0.58, p = 0.05) using the 12-point radiographic scale. All other radiographic, and ultrasonographic assessments failed to provide a statistically significantly difference using one-tailed t-tests (Tables 2–6). Similarly, there were no significant differences between the two treatment groups based upon the MRI evaluation using either the IW Dixon (p>0.05) or T1 TSE (P>0.05) images (Table 9).

**Table 4:** Summary of radiographic healing scores (5-point scale).

	4 week	7 week	10 week
**Saline**	1.47 (±0.65)	2.85 (±0.83)	3.58 (±0.72)
**PRP**	1.67 (±0.80)	2.93 (±0.85)	3.69 (±0.57)
**p-value**	0.15	0.36	0.26

**Table 5:** Summary of radiographic healing (12-point scale).

	4 week	7 week	10 week
**Saline**	3.00 (±1.95)	6.06 (±2.09)	8.58 (±2.53)
**PRP**	3.78 (±2.19)	7.07 (±2.30)	9.52 (±1.81)
**p-value**	0.08	0.04	0.05

**Table 6:** Summary of ultrasonographic assessment of cortical continuity (5-point scale).

	4 week	7 week	10 week
**Saline**	0.06 (±0.24)	1.12 (±0.86)	2.97 (±0.95)
**PRP**	0.11 (±0.32)	1.44 (±0.85)	3.30 (±0.67)
**p-value**	0.24	0.07	0.07

**Table 7:** Summary of ultrasonographic assessment of callus formation (5-point scale).

	4 week	7 week	10 week
**Saline**	1.64 (±0.42)	2.48 (±0.51)	3.25 (±0.54)
**PRP**	1.74 (±0.40)	2.59 (±0.33)	3.43 (±0.35)
**p-value**	0.16	0.17	0.08

**Table 8:** Summary of power doppler ultrasonographic assessment (5-point scale).

	4 week	7 week	10 week
**Saline**	1.45 (±0.52)	1.02 (±0.38)	0.76 (±0.37)
**PRP**	1.49 (±0.58)	1.08 (±0.36)	0.71 (±0.34)
**p-value**	0.38	0.24	0.31

**Table 9:** MRI assessment at 4 weeks.

	T1	T2 Dixon FS
**Saline**	223.1 (63.4)	172.0 (±64.5)
**PRP**	237.3 (69.7)	160.1 (±58.1)
**p-value**	0.41	0.46

Based upon the repeated measures analysis of covariance both time (4, 7, or 10 weeks post surgery; p<0.0001) and dog age (p<0.001) were significant predictors of radiographic healing using either the 5-point or 12-point scales. Body weight, gender, and treatment group (PRP versus control) all failed to have a significant effect (p>0.05) on radiographic assessment of healing. Similarly, time was a significant explanatory variable on ultrasonographic assessment of cortical healing (p<0.0001), callus formation (p<0.0001), and based upon power doppler analysis (p<0.0001). Age, bodyweight, gender, and treatment did not have a significant effect (p>0.05) on any of these ultrasonographic outcome measures. The explanatory variables and their associated p-values are shown in Table 10.

**Table 10:** P-values associated with each explanatory variable used in a repeated measures analysis of covariance.

	Time	Age	Weight	Gender	Treatment
**Radiographic Assessment**					
**5-point**	<0.0001	0.0003	0.66	0.31	0.92
**12-point**	<0.0001	<0.0001	0.17	0.18	0.22
**Ultrasonographic Assessment**					
**Cortical (5-point)**	<0.0001	0.47	0.55	0.99	0.14
**Callus (5-point)**	<0.0001	0.21	0.96	0.36	0.26
**Power Doppler**	<0.0001	0.12	0.08	0.62	0.54

## Discussion

There are few studies assessing the effects of PRP on long bone healing in dogs.[8, 25–27] One study showed a benefit in a radial gap model based upon radiographic and histologic assessments.[26] They used a population of dogs that was homogenous in body weight but varied in breed and there was no reporting on age of the animals or the effect of age on bone healing.[26] Conversely, another study failed to find a beneficial effect of PRP in combination with calcium phosphate granules in an ulnar defect in six beagles.[25, 26] A third study presumably failed to identify a benefit of PRP combined with hydroxyapatite in a radial gap stabilized with bone plates.[26, 27] Given these discrepant results the effects of different PRPs on long bone healing in dogs has remained unclear.

This study evaluated the effect of PRP on bone healing in a heterogeneous group of client-owned dogs with a commonly performed surgical procedure. The one-tailed t-tests demonstrated significantly greater 12-point radiographic healing at 7 and 10 weeks with use of PRP. However, the repeated measures analysis of covariance provided a more thorough analysis of explanatory variables that might have affected outcome using the radiographic and ultrasonographically acquired data. Those analyses showed that the only significant predictors of outcome were time since surgery and patient age. Even though there was not a significant difference between the two treatment groups with regards to body weight, gender, or age, the mean age of patients in the treatment group was slightly less than that in the control group. As a result, it seems likely that the small, and potentially clinically irrelevant, differences in radiographic scores seen between the two treatment groups at the 7 and 10 week time points was likely attributable to small differences in patient age rather than use of PRP. Accordingly, we reject our hypothesis that use of this specific PRP formulation would speed bone healing in dogs treated with a high tibial osteotomy.

With the aforementioned conclusion stated, it is worth considering the attributes and limitations of this study. Given the limitations of clinical research, this was a well-controlled study with a substantial number of dogs, the dogs treated in this study are similar in age and weight to those dogs typically treated with TPLO,[12] and that the statistically insignificant (or marginally significant) differences in radiographic healing scores between the two groups were small at all time points (Tables 4 and 5). Further, significant effects of time since surgery and age (radiographically) were detected for all radiographic (5-point and 12-point) and ultrasonographic (cortical, callus, and power Doppler) measures of healing. Given that both time since surgery and animal age are biologically plausible explanatory variables affecting bone healing, these results provide some evidence that the outcome measures were valid in addition to being derived from clinically relevant modalities. In turn, we do not believe that the outcome measures were insensitive or use of a greater number of dogs would elucidate a clinically relevant benefit of this type of PRP for dogs treated with TPLO. This conclusion is based in part on the opinion that differences in radiographic healing scores of ≤ 0.2 (5-point scale) or ≤1.01 (12-point scale) do not justify the use of this PRP in dogs treated with TPLO. However, it is possible with inclusion of a greater number of dogs that the effect of treatment could become statistically significant, albeit it is difficult to assess the number of dogs needed for a multivariate analysis that accounts for repeated measures over time and covariates such as age, body weight, and body condition score.

In addition to the possibility of a type II statistical error, there are several possible reasons that this study failed to provide evidence of augmented bone healing using PRP. One possible explanation for the lack of a significant treatment effect is that the elution of anabolic growth factors from activated PRP gels are transient.[28] Visser *et al*. (2010) demonstrated that elution of transforming growth factor-β1 eluted from canine platelet-rich fibrin membranes and matrices was greatest within the first 24 hours and was essentially negligible by 5 days.[16] Using the specific PRP production and activation methodology as was used in this study, we similarly identified that elution of both TGF-β1 and PDGF-BB was greatest in the first 24 hours and substantially lower by day 3 in vitro and virtually negligible by day 5 (data not shown). As a result, the possible benefits of PRP on bone regeneration may be hampered by fleeting delivery of anabolic growth factors.

A second possible reason that this study did not provide evidence of enhanced bone healing with use of PRP is that we performed a high tibial osteotomy that results in substantial bone apposition in a group of healthy patients scheduled for an elective surgery. With substantial bone apposition in a healthy groups of patients osseous healing proceeded uneventfully in the control group. Further, the lack of a gap probably mitigates identification of enhanced bone regeneration, which can be visualized more easily within a gap or defect.[26] Likewise, we obtained no histologic or mechanical data to assess the effects of PRP as these were client-owned dogs. Although these latter outcomes measures may increase the possibility of detecting a significant difference between groups they are not clinically applicable, whether in dogs or people.

A third possible explanation for an inability to identify a treatment effect is that we used a heterogeneous population of study subjects. This characteristic of the study potentially decreased sensitivity in comparison to if an experimental study were performed in which breed, age, weight, body condition score, and gender were uniform using a population of purpose-bred research dogs. Likewise, although the same post-operative activity restriction was advised for all dogs included in this study there is likely variability in post-operative activity of client-owned dogs that could be more rigorously standardized with purpose-bred research dogs. However, the inclusion of clinically-relevant heterogeneity is also a desirable characteristic of this study as it provided a relevant representation of clinical practice in which heterogeneity exists, whether treating canine or human patients

A fourth possible reason for failure to augment bone healing could pertain to the specific PRP that was used in this study. At least two studies have demonstrated that the concentration of calcium in collagen gels and PRP scaffolds affect mouse osteoblast and human umbilical stem cell viability with higher concentrations of calcium having negative effects on cellular viability.[29, 30] Specifically, use of 10% calcium chloride solution combined with PRP in a 1:10 ratio were associated with less umbilical stem cell viability than use of a lower concentration (2.5%) of calcium chloride with the PRP scaffold.[30] In this study, we used 10% calcium chloride combined with thrombin in a 1:10 ratio for PRP activation, similar to the higher concentration used in the aforementioned study.[30] In addition, bovine thrombin is known to induce immune or inflammatory reactions in people and rodents.[31] The use of bovine thrombin in this study may have induced an immune or inflammatory response that could have precluded benefit with use of the PRP. Further, high platelet concentrations in PRP are not always beneficial and some studies have shown that higher platelet or growth factor concentrations are detrimental to tendon and bone healing.[32, 33] We used PRP with a high platelet concentration and this may have precluded any benefit of such treatment.

Although the results of this study fail to demonstrate a benefit of bone healing, it is important to not extrapolate these results to conclude that PRP cannot have any positive effect on bone regeneration. Conclusions made based upon studies of PRP should remain specific to the species studied, the medical condition or model in which the investigation was performed, the characteristics of the PRP, and with consideration to the outcome measures used. While understanding those guidelines and the inability to draw definitive conclusions that extrapolate to other species, but also considering the possible translational relevance of this study, these data do not support investigation of a similar human PRP in conjunction with a well-apposed and rigidly stabilized osteotomy in people, such as a closing-wedge high tibial osteotomy. This latter conclusion is obviously tenuous because this study was performed in dogs and not people. However, these results and this latter conclusion is at least consistent with numerous clinical studies in people that fail to identify a positive effect of PRP on bone healing, even when open fracture gaps and open osteotomies are considered.[7] Rather, further studies should likely be performed in relevant models or human clinical populations in which bone loss, fracture gaps, or delayed union are present so as to optimize the likelihood of identifying a treatment effect.

## Supporting information

S1 Table Ultrasonography scores. This file includes all the ultrasonography scores for all dogs included in this study including scores of cortical bone healing, assessment of healing callus, and power doppler assessment.
(XLSX)

S2 Table MRI scores. This file includes all collected MRI data.
(XLSX)

S3 Table Radiographic scores. This file includes all radiographic scores for assessment of fracture healing.
(XLSX)

S1 Fig This file includes the completed ARRIVE guidelines checklist. (PDF)
